# Chemical Composition, Antibacterial, and Anti-Inflammatory Activities of Essential Oils from Flower, Leaf, and Stem of *Rhynchanthus beesianus*

**DOI:** 10.1155/2021/5562461

**Published:** 2021-04-29

**Authors:** Qi Chen, Xiaoge Zhao, Tingya Lu, Yao Yang, Yi Hong, Minyi Tian, Ying Zhou

**Affiliations:** ^1^Key Laboratory of Plant Resource Conservation and Germplasm Innovation in Mountainous Region (Ministry of Education), Collaborative Innovation Center for Mountain Ecology & Agro-Bioengineering (CICMEAB), Institute of Agro-Bioengineering, College of Life Sciences, Guizhou University, Guiyang 550025, China; ^2^National & Local Joint Engineering Research Center for the Exploition of Homology Resources of Southwest Medicine and Food, Guizhou University, Guiyang, China; ^3^College of Pharmacy, Guizhou University of Traditional Chinese Medicine, Guiyang 550025, China

## Abstract

*Rhynchanthus beesianus* is a medicinal, ornamental, and edible plant, and its essential oil has been used as an aromatic stomachic in China. In this study, the chemical constituents, antibacterial, and anti-inflammatory properties of flower essential oil (F-EO), leaf essential oil (L-EO), and stem essential oil (S-EO) of *R. beesianus* were investigated for the first time. According to the GC-FID/MS assay, the F-EO was mainly composed of bornyl formate (21.7%), 1,8-cineole (21.6%), borneol (9.7%), methyleugenol (7.7%), *β*-myrcene (5.4%), limonene (4.7%), camphene (4.5%), linalool (3.4%), and *α*-pinene (3.1%). The predominant components of L-EO were bornyl formate (33.9%), borneol (13.2%), 1,8-cineole (12.1%), methyleugenol (8.0%), camphene (7.8%), bornyl acetate (6.2%), and *α*-pinene (4.3%). The main components of S-EO were borneol (22.5%), 1,8-cineole (21.3%), methyleugenol (14.6%), bornyl formate (11.6%), and bornyl acetate (3.9%). For the bioactivities, the F-EO, L-EO, and S-EO exhibited significant antibacterial property against *Bacillus subtilis*, *Enterococcus faecalis*, *Staphylococcus aureus*, *Proteus vulgaris*, *Pseudomonas aeruginosa*, and *Escherichia coli* with the inhibition zones (7.28–9.69 mm), MIC (3.13–12.50 mg/mL), and MBC (6.25–12.50 mg/mL). Besides, the F-EO, L-EO, and S-EO significantly inhibited the production of proinflammatory mediator nitric oxide (NO) (93.15–94.72%) and cytokines interleukin-6 (IL-6) (23.99–77.81%) and tumor necrosis factor-*α* (TNF-*α*) (17.69–24.93%) in LPS-stimulated RAW264.7 cells at the dose of 128 *μ*g/mL in the absence of cytotoxicity. Hence, the essential oils of *R. beesianus* flower, leaf, and stem could be used as natural antibacterial and anti-inflammatory agents with a high application potential in the pharmaceutical and cosmetic fields.

## 1. Introduction

Essential oils are a mixture of natural volatile compounds from different parts of plants and have been widely used in cosmetic, perfume, agriculture, food, and medicine fields [1, 2]. Essential oils have been used as complementary and alternative therapies to treat cancer, high blood pressure, pain, rheumatoid arthritis, and so on [3]. The side effects of synthetic drugs, the high resistance rate of pathogen strains, and the limitations of existing antibiotics/drugs have motivated people to seek and use alternative or complementary therapies, including the use of essential oils [3, 4]. The family Zingiberaceae consists of approximately 52 genera and 1600 species, many of which are rich in essential oils [5, 6]. According to the previous studies, the essential oils of Zingiberaceae plants have a great variety of pharmacological activities, such as antimicrobial, anti-inflammatory, insecticidal, antiulcer, antiallergic, analgesic, antimutagenic, anticancer, and immunomodulatory properties [7–11].

*Rhynchanthus* J. D. Hooker is a small genus of Zingiberaceae, with about four species distributed in Indonesia, Myanmar, and Southern China [12, 13]. *Rhynchanthus beesianus* W. W. Smith is a perennial herb, cultivated as a medicinal, edible, and ornamental plant in Myanmar and Southern China [13, 14]. *R. beesianus* is a wild edible spice, and its tender leaf and rhizome are used as vegetables in Yunnan Province, China. *R. beesianus* flower with brilliant color and peculiar brush shape is used as a fresh cut flower. Its rhizome has been used as an aromatic stomachic in traditional Chinese medicine to treat stomachache and indigestion [15–17]. Additionally, the essential oils from *R. beesianus* have been used as an aromatic stomachic in China [17]. According to the previous study, the essential oil of *R. beesianus* rhizome was mainly composed of 1,8-cineole (47.6%), borneol (15.0%), methyleugenol (11.2%), and bornyl formate (7.6%) and was found to possess antibacterial, anti-inflammatory, *α*-glucosidase, and acetylcholinesterase activity inhibitory properties [18]. *R. beesianus* mainly relies on the vegetative propagation of rhizome for population expansion. Only harvesting the aerial parts (flower, leaf, and stem) of *R. beesianus* can reduce its damage, which is conducive to its sustainable use. However, there are no reports on the chemical components and antibacterial and anti-inflammatory properties of essential oils from *R. beesianus* flower, leaf, and stem.

## 2. Materials and Methods

### 2.1. Plant Material

The flower, leaf, and stem of *R. beesianus* were collected in July 2019 from Guangxi Province of China. Plant materials were identified by Prof. Guoxiong Hu of Guizhou University. Voucher specimens were kept at the National & Local Joint Engineering Research Center for the Exploition of Homology Resources of Southwest Medicine and Food, Guizhou University (Voucher No: RB-20190712).

### 2.2. Essential Oils' Extraction

The fresh, finely chopped *R. beesianus* flower, leaf, and stem (1.0 kg) were separately extracted by hydrodistillation using a Clevenger-type apparatus. After 4 h, the flower, leaf, and stem essential oils were separately obtained and dried over anhydrous Na_2_SO_4_. Then, all essential oils were kept at 4°C in the amber bottle for further tests.

### 2.3. Chromatographic Analysis

The essential oils were analyzed by an Agilent 6890 gas chromatograph (GC) equipped with an HP-5MS capillary column (60 m × 0.25 mm, 0.25 *μ*m film thickness) and a flame ionization detector (FID) (Agilent Technologies Inc., CA, USA). The split ratio was 1 : 20 (injection volume: 1 *μ*L) with helium as carrier gas (1 mL/min). The GC oven temperature was as follows: held at 70°C (2 min), 2°C/min to 180°C (55 min), 10°C/min to 310°C (13 min), and kept at 310°C (4 min). The GC-MS analysis was carried out using an Agilent 6890 gas chromatograph equipped with an Agilent 5975C mass selective detector. The Agilent 6890 gas chromatograph (GC) coupled to an Agilent 5975C mass selective detector (MS) was used to identify the chemical composition of the essential oils. The parameters of GC and capillary column were the same as in GC-FID. The MS was operated in the electron ionization mode at 70 eV and the mass range (*m*/*z* 29 to 500). The ion source temperature and interface temperature were 230°C and 280°C, respectively. The relative percentage of chemical constituents was determined by the peak area normalization method. A series of n-alkanes (C_8_–C_30_) were injected to calculate the retention index. The components of the essential oils were identified by comparison of their mass spectrum and calculated retention index and with those listed in Wiley 275 and NIST 17 databases.

### 2.4. Antimicrobial Activity

#### 2.4.1. Bacterial Strains

The antibacterial activity was evaluated against *Bacillus subtilis* ATCC 6633, *Enterococcus faecalis* ATCC 19433, *Staphylococcus aureus* ATCC 6538P, *Proteus vulgaris* ACCC 11002, *Pseudomonas aeruginosa* ATCC 9027, and *Escherichia coli* CICC 10389.

#### 2.4.2. Agar Well Diffusion Assay

The inhibition zone diameters were measured according to the agar well diffusion method with marginal modification [19]. The essential oils and streptomycin (positive control) were separately dissolved in ethyl acetate (100 mg/mL) and distilled water solution (100 *μ*g/mL). 100 *μ*L of bacterial suspensions (10^6^ CFU/mL) was evenly inoculated on the Mueller-Hinton agar plate. Then, filter paper discs of 6 mm diameter containing sample solution (20 *μ*L) were added. After 24 h incubation at 37°C, the inhibition zone diameters were measured.

#### 2.4.3. Determination of MIC and MBC

The minimal inhibitory concentration (MIC) and minimal bactericidal concentration (MBC) values were assayed by our previously published microplate dilution method [20]. Briefly, 100 *μ*L of bacterial suspension and twofold serially diluted sample solution (100 *μ*L) were added to each well at a final density of 10^5^ CFU/mL and incubated at 37°C for 24 h. Subsequently, resazurin solutions (20 *μ*L, 0.1 mg/mL) were added to each well. After 2 h incubation at 37°C in the dark, the MIC values were determined as the minimum sample concentration without color change. For the determination of the MBC values, 10 *μ*L of samples from the wells without color change was subcultured on Mueller-Hinton agar medium. After 24 h incubation at 37°C, the MBC values were determined as the minimum sample concentration without bacterial growth.

### 2.5. Anti-Inflammatory Activity

#### 2.5.1. Cytotoxic Assay

The cytotoxicity was evaluated on murine fibroblast cells (L929) and murine macrophages (RAW264.7) by the MTT assay with slight modification [21]. The L929 and RAW264.7 cells were separately maintained in RPMI 1640 medium and DMEM medium (10% fetal bovine serum, 2 mM glutamine, 100 *μ*g/mL streptomycin, and 100 U/mL penicillin) and incubated in a humidified incubator at 37°C with 5% CO_2_ atmosphere. 100 *μ*L of cell suspensions was added to each well at a density of 2 × 10^4^ cells per well. After 24 h incubation, twofold serially diluted essential oil solutions (100 *μ*L) were added to each well and incubated for 24 h. Subsequently, 10 *μ*L of MTT solution (5 mg/mL in PBS) was added and incubated for 4 h. After discarding the supernate, DMSO (150 *μ*L) was added to each well to dissolve the formazan crystal. The absorbance was recorded at 490 nm using a Varioskan Lux Multimode microplate reader (Thermo Fisher Scientific, USA).

#### 2.5.2. Morphology Assay and Measurement of NO, IL-6, and TNF-*α*

100 *μ*L of RAW264.7 cell suspensions was added to each well at a density of 2 × 10^4^ cells per well and incubated for 24 h. After discarding the medium, twofold serially diluted essential oil solutions (100 *μ*L) were added and incubated for 2 h. Subsequently, lipopolysaccharide solutions (LPS, 100 *μ*L) were added to each well at a final concentration of 1 *μ*g/mL and incubated for 24 h. Morphological changes were recorded using a Leica DMi8 inverted microscope (Leica Microsystems, Germany). Then, the supernatants were collected and centrifuged. The accumulation of NO in the culture supernatant was determined by a colorimetric NO detection kit following the manufacturer's instructions (Nanjing Jiancheng Bioengineering Institute, Nanjing, China). Dexamethasone (DXM, 20 *μ*g/mL) was used as a positive reference. The secretion of IL-6 and TNF-*α* was assayed by ELISA kits in accordance with the manufacturer's instructions (MultiSciences Biotech Co., Ltd., Hangzhou, China).

### 2.6. Statistical Analysis

All experiments were performed independently at least three times, and the results were expressed as the means ± SD. SPSS software (version 19.0) was used for statistical analysis. Data were compared by one-way analysis of variance (ANOVA) using Fisher's LSD post hoc tests. Differences were considered significant at the *p* < 0.05 level.

## 3. Results and Discussion

### 3.1. Chemical Composition

**Figure 1:**
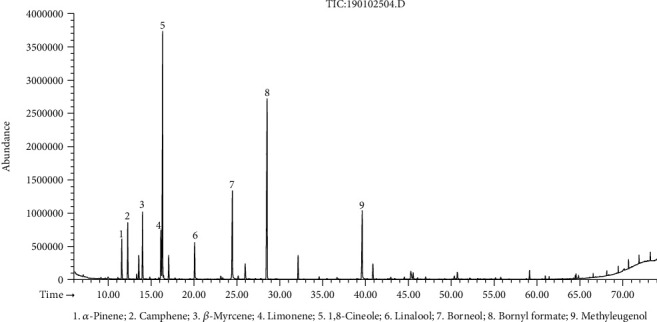
GC-MS chromatogram of *R. beesianus* F-EO.

**Figure 2:**
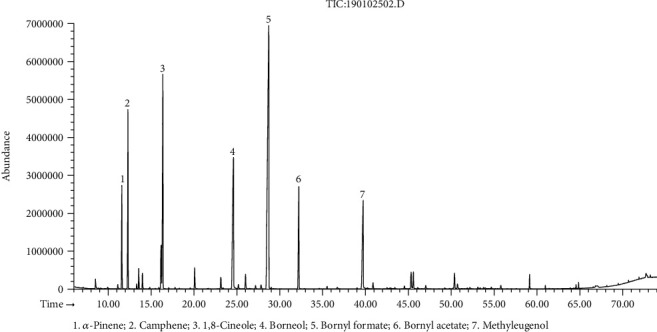
GC-MS chromatogram of *R. beesianus* L-EO.

**Figure 3:**
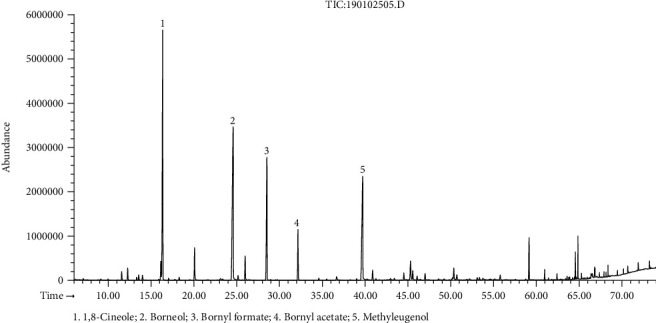
GC-MS chromatogram of *R. beesianus* S-EO.

The hydrodistillation of fresh flower, leaf, and stem of *R. beesianus* separately yielded essential oils at 0.21% (*w*/*w*), 0.47% (*w*/*w*), and 0.94% (*w*/*w*) on a fresh weight basis. The GC-FID/MS analysis showed the identification of forty-six, forty-four, and sixty-three compounds accounting for 98.6%, 98.7%, and 96.0% of the total oil content of flower, leaf, and stem, respectively (Table 1). *R. beesianus* F-EO was mainly composed of bornyl formate (21.7%), 1,8-cineole (21.6%), borneol (9.7%), methyleugenol (7.7%), *β*-myrcene (5.4%), limonene (4.7%), camphene (4.5%), linalool (3.4%), and *α*-pinene (3.1%) (Figure 1). The predominant components of L-EO were bornyl formate (33.9%), borneol (13.2%), 1,8-cineole (12.1%), methyleugenol (8.0%), camphene (7.8%), bornyl acetate (6.2%), and *α*-pinene (4.3%) (Figure 2). The S-EO was mainly composed of borneol (22.5%), 1,8-cineole (21.3%), methyleugenol (14.6%), bornyl formate (11.6%), and bornyl acetate (3.9%) (Figure 3). In our previous study, the yield of *R. beesianus* rhizome oil was 0.22% (*w*/*w*), and its predominance components were 1,8-cineole (47.6%), borneol (15.0%), methyleugenol (11.2%), and bornyl formate (7.6%) [18]. *R. beesianus* stem had the highest essential oil yield, as compared with the flower, leaf, and rhizome. Hence, the observed difference in the yield and composition of the essential oils could be attributed to the part of the plant used.

### 3.2. Antimicrobial Activity

The antibacterial properties of essential oils were qualitatively determined by the inhibition zone diameters (Table 2) and quantitatively evaluated by the minimal inhibitory concentration (MIC) and minimal bactericidal concentration (MBC) values (Table 3). Streptomycin was used as a positive reference. The *R. beesianus* F-EO, L-EO, and S-EO showed broad-spectrum antibacterial effect with DIZ values between 7.28 and 9.69 mm against *Bacillus subtilis* (MIC: 12.50 mg/mL, MBC: 12.50 mg/mL), *Enterococcus faecalis* (MIC: 3.13–6.25 mg/mL, MBC: 6.25 mg/mL), *Staphylococcus aureus* (MIC: 6.25–12.50 mg/mL, MBC: 12.50 mg/mL), *Proteus vulgaris* (MIC: 3.13 mg/mL, MBC: 12.50 mg/mL), *Pseudomonas aeruginosa* (MIC: 6.25 mg/mL, MBC: 12.50 mg/mL), and *Escherichia coli* (MIC: 3.13–6.25 mg/mL, MBC: 6.25–12.50 mg/mL). In previous studies, the antibacterial activity of predominance components, such as borneol, 1,8-cineole, methyleugenol, *β*-myrcene, limonene, camphene, and *α*-pinene, has been demonstrated [22–27]. Hence, these major constituents could explain the significant antibacterial properties of *R. beesianus* F-EO, L-EO, and S-EO. These results suggest that *R. beesianus* F-EO, L-EO, and S-EO can be used as a natural source of antibacterial agents for the pharmaceutical and cosmetic industries.

### 3.3. Anti-Inflammatory Activity

**Figure 4:**
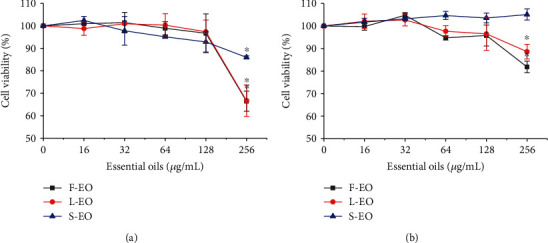
Effects of F-EO, L-EO, and S-EO on cell viability of RAW264.7 (a) and L929 (b) cells. The data were presented as mean ± standard deviation (SD) of three independent experiments. ^∗^*p* < 0.05, compared to untreated control group cells.

**Figure 5:**
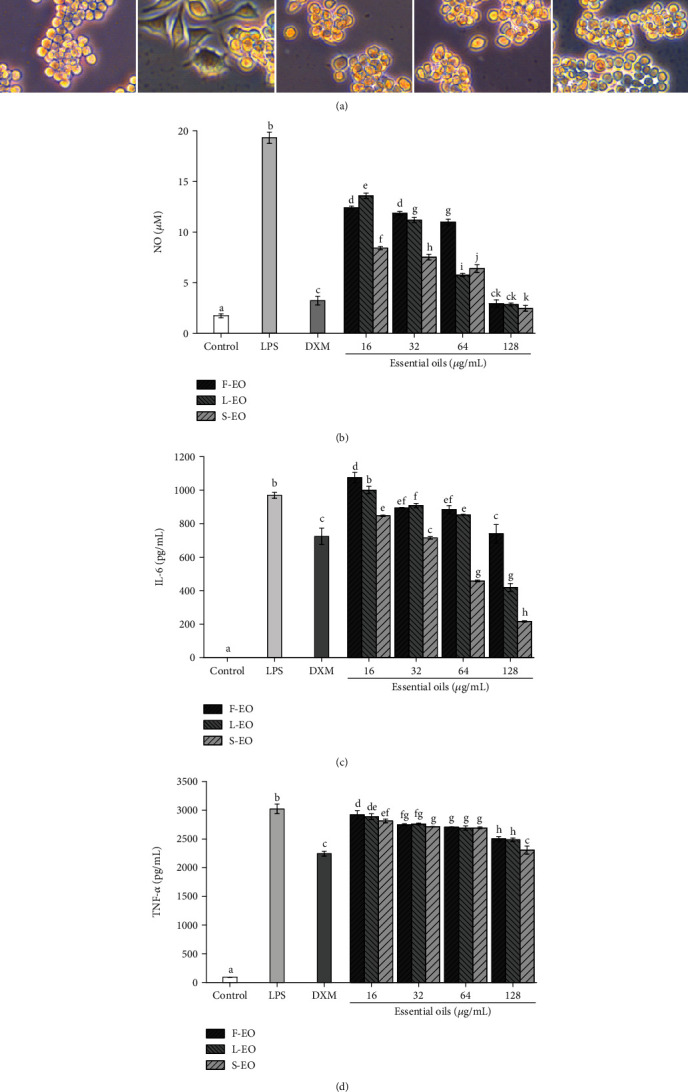
Effects of F-EO, L-EO, and S-EO on the LPS-induced RAW264.7 cell morphology (128 *μ*g/mL) (a), NO production (b), and secretion of IL-6 (c) and TNF-*α* (d). The results of at least three independent experiments were expressed as mean ± SD values. (b-d) Different letters above bars represent a significant difference (*p* < 0.05).

The inhibitory effects of F-EO, L-EO, and S-EO on the proinflammatory mediator (NO) and cytokines (IL-6 and TNF-*α*) were investigated in the lipopolysaccharide- (LPS-) stimulated RAW264.7 macrophages. According to the MTT assay, all essential oils revealed no significant cytotoxic effect on RAW264.7 and L929 cells at a dose of 16-128 *μ*g/mL in comparison with the untreated control cells (*p* > 0.05) (Figure 4). Hence, the dose of 16-128 *μ*g/mL was used in subsequent experiments. As shown in Figure 5(a), LPS-induced RAW264.7 macrophages became irregular in shape and increased in size compared to those in the control group. Compared with the LPS-induced group, RAW264.7 cells in the F-EO, L-EO, and S-EO at doses of 128 *μ*g/mL treated group exhibited relatively smooth surfaces. The accumulation of NO in the culture supernatant was detected by a colorimetric NO detection kit using dexamethasone (DXM, 20 *μ*g/mL) as a positive reference. All essential oils dose-dependently inhibited NO accumulation (Figure 5(b) and Table 4). In particular, compared with the LPS group (19.31 ± 0.56 *μ*M), the F-EO, L-EO, and S-EO (128 *μ*g/mL) significantly decreased NO production by 2.93 ± 0.38, 2.84 ± 0.15, and 2.46 ± 0.28 *μ*M, respectively. The inhibitory ratios of F-EO (93.15 ± 1.36%), L-EO (93.90 ± 1.34%), and S-EO (94.72 ± 1.34%) at doses of 128 *μ*g/mL were comparable to DXM (91.44 ± 1.70%, 3.23 ± 0.42 *μ*M). The secretion of IL-6 and TNF-*α* was assayed by ELISA kits. All essential oils potently suppressed the secretion of IL-6 in LPS-induced RAW264.7 macrophages, and the inhibitory ratios of S-EO at 64 *μ*g/mL (52.82 ± 0.26%) and 128 *μ*g/mL (77.81 ± 0.20%) and L-EO at 128 *μ*g/mL (56.98 ± 2.06%) were exceeded that of DXM (25.69 ± 4.39% at 20 *μ*g/mL) (Figure 5(c) and Table 4). Besides, compared with the LPS group (3024.36 ± 85.32 pg/mL), the secretion of TNF-*α* was significantly decreased by F-EO (2504.25 ± 39.23 pg/mL), L-EO (2485.88 ± 29.91 pg/mL), and S-EO (2308.23 ± 69.09 pg/mL) at doses of 128 *μ*g/mL (Figure 5(d)). As shown in Table 4, the inhibitory ratio of TNF-*α* of S-EO (24.93 ± 2.23%) was equivalent to that of DXM (27.07 ± 0.42%). The proinflammatory cytokines (IL-6 and TNF-*α*) and mediator (NO) play key roles in inflammation disorders, and reducing their release is a promising strategy to treat inflammation-related diseases [28]. In our previous study, *R. beesianus* rhizome essential oil (128 *μ*g/mL) significantly inhibited the production of NO (92.73 ± 1.50%), IL-6 (61.08 ± 0.13%), and TNF-*α* (20.29 ± 0.17%) in LPS-induced RAW264.7 cells [18]. Compared with the essential oils of *R. beesianus* flower, leaf, and rhizome, the essential oil of stem showed the strongest anti-inflammatory activity. The main components in the essential oils, such as 1,8-cineole, methyleugenol, borneol, *α*-pinene, linalool, limonene, *β*-myrcene, and bornyl acetate, have been demonstrated to have anti-inflammatory activity [29–35]. Hence, the anti-inflammatory activity of F-EO, L-EO, and S-EO may be due to these predominant constituents. These results suggest that *R. beesianus* F-EO, L-EO, and S-EO can provide natural anti-inflammatory agents for the pharmaceutical and cosmetic industries.

## 4. Conclusion

To our knowledge, this is the first report on the chemical constituents and bioactivities of essential oils from *R. beesianus* flower, leaf, and stem. Forty-six, forty-four, and sixty-three compounds were identified in the F-EO, L-EO, and S-EO by using GC-FID/MS, respectively. The F-EO, L-EO, and S-EO exhibited significant antibacterial property against *Bacillus subtilis*, *Enterococcus faecalis*, *Staphylococcus aureus*, *Proteus vulgaris*, *Pseudomonas aeruginosa*, and *Escherichia coli*. Besides, the F-EO, L-EO, and S-EO significantly inhibited the production of proinflammatory mediator NO and cytokines (IL-6 and TNF-*α*) in LPS-stimulated RAW264.7 cells in the absence of cytotoxicity. In particular, the essential oil of the stem showed the highest yield and anti-inflammatory activity. Hence, the essential oils of *R. beesianus* flower, leaf, and stem could be regarded as antibacterial and anti-inflammatory natural products with a high application potential in the pharmaceutical and cosmetic fields.

## Figures and Tables

**Table 1:** Chemical composition of *R. beesianus* F-EO, L-EO, and S-EO.

Compounds^a^	RI^b^	RI^c^	% area			Identification^d^
			F-EO	L-EO	S-EO	
Octane	800	800	0.1	tr^e^	0.1	MS, RI
*cis*-3-Hexen-1-ol	857	850	—	0.5	—	MS, RI
Ethylbenzene	855	863	—	—	tr^e^	MS, RI
*m*-Xylene	866	871	—	—	0.1	MS, RI
*o*-Xylene	887	900	—	—	0.1	MS, RI
Tricyclene	925	926	0.1	0.2	tr^e^	MS, RI
*α*-Thujene	929	928	0.1	—	tr^e^	MS, RI
*α*-Pinene	937	937	3.1	4.3	0.6	MS, RI
Camphene	952	952	4.5	7.8	0.8	MS, RI
Sabinene	974	976	0.4	0.2	0.2	MS, RI
*β*-Pinene	979	982	2.0	0.9	0.4	MS, RI
*β*-Myrcene	991	991	5.4	0.7	0.4	MS, RI
*α*-Phellandrene	1005	1008	0.2	0.1	tr^e^	MS, RI
*α*-Terpinene	1017	1020	0.1	tr^e^	—	MS, RI
*p*-Cymene	1023	1027	0.1	0.1	0.1	MS, RI
Limonene	1030	1031	4.7	2.8	1.7	MS, RI
1,8-Cineole	1032	1035	21.6	12.1	21.3	MS, RI
*cis*-Ocimene	1038	1037	0.3	—	—	MS, RI
*α*-Ocimene	1047	1047	2.0	tr^e^	0.1	MS, RI
*γ*-Terpinene	1060	1061	0.1	0.1	0.1	MS, RI
*cis*-4-Thujanol	1070	1069	0.1	tr^e^	0.2	MS, RI
*cis*-Linalool oxide	1074	1074	0.0	—	—	MS, RI
Terpinolene	1088	1092	0.1	tr^e^	tr^e^	MS, RI
Linalool	1099	1101	3.4	1.0	2.4	MS, RI
*trans-*Verbenol	1144	1148	—	—	0.1	MS, RI
Camphor	1145	1149	0.3	0.6	0.1	MS, RI
Camphene hydrate	1148	1153	—	0.1	—	MS, RI
Borneol	1167	1171	9.7	13.2	22.5	MS, RI
Terpinen-4-ol	1177	1181	0.3	0.3	0.3	MS, RI
*α*-Terpineol	1190	1194	1.5	0.9	1.9	MS, RI
Bornyl formate	1226	1234	21.7	33.9	11.6	MS, RI
Isobornyl formate	1232	1240	tr^e^	0.1	tr^e^	MS, RI
Carvone	1242	1248	tr^e^	—	—	MS, RI
Isopentyl hexanoate	1252	1250	0.1	—	0.0	MS, RI
Bornyl acetate	1284	1290	2.3	6.2	3.9	MS, RI
*γ*-Pyronene	1338	1342	—	0.1	0.1	MS, RI
Daucene	1381	1385	—	—	tr^e^	MS, RI
*β*-Elemen	1394	1395	0.1	tr^e^	0.1	MS, RI
Methyleugenol	1402	1407	7.7	8.0	14.6	MS, RI
*α*-Gurjunene	1409	1417	—	tr^e^	0.1	MS, RI
Caryophyllene	1419	1427	1.5	0.3	0.8	MS, RI
Aromandendrene	1440	1456	0.1	—	—	MS, RI
*cis*-*β*-Farnesene	1445	1459	0.1	—	—	MS, RI
Humulene	1454	1461	0.2	—	0.1	MS, RI
*epi*-*β*-Caryophyllene	1466	1469	0.1	0.1	0.1	MS, RI
*α*-Curcumene	1483	1487	0.3	0.2	0.6	MS, RI
Methylisoeugenol	1492	1499	1.1	1.1	2.0	MS, RI
Bicyclogermacrene	1499	1504	0.6	1.0	0.7	MS, RI
*β*-Bisabolene	1509	1513	0.1	0.1	0.2	MS, RI
Sesquicineole	1516	1519	—	—	tr^e^	MS, RI
*δ*-Cadinene	1524	1529	0.2	0.2	0.5	MS, RI
2-(4-Ethenyl-4-methyl-3-prop-1-en-2-ylcyclohexyl)propan-2-ol	1549	1555	—	—	0.1	MS, RI
Nerolidol	1564	1567	0.1	tr^e^	0.1	MS, RI
Palustrol	1568	1577	—	—	tr^e^	MS, RI
Germacren D-4-ol	1574	1583	—	—	0.2	MS, RI
Spathulenol	1576	1586	0.3	0.9	1.1	MS, RI
Caryophyllene oxide	1581	1592	0.7	0.3	0.4	MS, RI
Ledol	1607	1612	—	0.1	—	MS, RI
Isospathulenol	1638	1646	—	—	0.2	MS, RI
*β*-Eudesmol	1649	1660	—	—	0.1	MS, RI
*α*-Cadinol	1653	1663	—	0.1	0.1	MS, RI
Ambrial	1809	1815	0.7	0.1	0.5	MS, RI
Hexahydrofarnesyl acetone	1844	1850	—	—	tr^e^	MS, RI
Isophytol	1948	1953	—	—	0.1	MS, RI
Pimaradiene	1996	1990	0.2	0.1	1.0	MS, RI
(*E*)-15,16-Dinorlabda-8 (17),11-dien-13-one	1994	2009	—	—	1.6	MS, RI
Geranyl linallol	2034	2039	—	—	0.2	MS, RI
Abietatriene	2054	2086	—	—	0.1	MS, RI
Phytol	2114	2122	—	—	0.5	MS, RI
Coronarin E	2136	2159	—	—	0.3	MS, RI
Tricosane	2300	2299	—	—	0.4	MS, RI
Pentacosane	2500	2498	—	—	0.1	MS, RI
Total			98.6	98.7	96.0	

^a^Compounds were listed in the order of their elution on the HP-5MS column. ^b^Retention index (RI) on the HP-5MS column, using a homologous series of n-alkanes (C_8_–C_30_) as references. ^c^RI in Wiley 275 and NIST 17 mass spectral libraries. ^d^Identification: MS by comparison with Wiley 275 and NIST 17 mass spectrum libraries; RI by comparison of retention index with those reported in NIST 17 and Wiley 275 libraries. -: not detected. ^e^tr: trace (trace < 0.01%).

**Table 2:** The inhibition zone diameters of *R. beesianus* F-EO, L-EO, and S-EO^a^.

Microorganisms	The inhibition zone diameters (mm)			
	F-EO	L-EO	S-EO	Streptomycin
*Gram positive*				
*Bacillus subtilis* ATCC 6633	8.29 ± 0.26	8.92 ± 0.51	7.81 ± 0.43	19.01 ± 0.40
*Enterococcus faecalis* ATCC 19433	8.46 ± 0.61	9.36 ± 0.68	8.38 ± 0.41	7.57 ± 0.43
*Staphylococcus aureus* ATCC 6538P	7.96 ± 0.34	8.12 ± 0.18	8.39 ± 0.53	18.41 ± 0.45
*Gram negative*				
*Proteus vulgaris* ACCC 11002	8.91 ± 0.22	9.29 ± 0.43	8.67 ± 0.23	15.08 ± 0.43
*Pseudomonas aeruginosa* ATCC 9027	7.82 ± 0.65	7.28 ± 0.15	7.38 ± 0.16	10.06 ± 0.60
*Escherichia coli* CICC 10389	7.87 ± 0.31	9.69 ± 0.59	8.29 ± 0.32	18.41 ± 0.70

^a^Diameter of the inhibition zone includes diameter of the disk (6 mm). Essential oil solutions were dissolved with ethyl acetate (tested volume: 20 *μ*L, 100 mg/mL); streptomycin distilled water solution (tested volume: 20 *μ*L, 100 *μ*g/mL) was used as a positive control.

**Table 3:** The MIC and MBC values of *R. beesianus* F-EO, L-EO, and S-EO^a^.

Microorganism	F-EO (mg/mL)		L-EO (mg/mL)		S-EO (mg/mL)		Streptomycin (*μ*g/mL)	
	MIC	MBC	MIC	MBC	MIC	MBC	MIC	MBC
*Gram positive*								
*B. subtilis*	12.50	12.50	12.50	12.50	12.50	12.50	0.39	0.78
*E. faecalis*	6.25	6.25	3.13	6.25	3.13	6.25	12.50	25.00
*S. aureus*	12.50	12.50	12.50	12.50	6.25	12.50	0.78	1.56
*Gram negative*								
*P. vulgaris*	3.13	12.50	3.13	12.50	3.13	12.50	0.39	1.56
*P. aeruginosa*	6.25	12.50	6.25	12.5	6.25	12.50	3.13	12.50
*E. coli*	6.25	12.50	3.13	6.25	3.13	12.50	0.19	1.56

^a^ MIC: minimal inhibitory concentration; MBC: minimal bactericidal concentration; streptomycin as a positive control.

**Table 4:** NO, IL-6, and TNF-*α* inhibition effects of F-EO, L-EO, and S-EO on LPS-induced RAW264.7 cells.

Treatment	Dose (*μ*g/mL)	Inhibition (%)		
		NO	IL-6	TNF-*α*
DXM	20	91.44 ± 1.70^a^	25.69 ± 4.39^a^	27.07 ± 0.42^a^

F-EO	16	38.60 ± 1.56^b^	−10.45 ± 4.73^b^	5.10 ± 2.97^b,c^
	32	42.37 ± 2.47^c^	7.80 ± 1.34^c^	9.40 ± 3.22^b,c,d^
	64	46.19 ± 1.23^d^	9.21 ± 2.12^c^	10.75 ± 2.70^b,d^
	128	93.15 ± 1.36^a,e^	23.99 ± 6.34^a^	17.69 ± 3.74^f^

L-EO	16	32.14 ± 0.80^f^	−2.83 ± 1.80^d^	4.57 ± 1.11^c^
	32	45.89 ± 1.86^d^	6.74 ± 1.73^c^	8.94 ± 3.19^b,c,d^
	64	77.02 ± 0.80^g^	11.99 ± 1.33^c^	11.34 ± 3.85^d^
	128	93.90 ± 1.34^a,e^	56.98 ± 2.06^e^	18.32 ± 3.40^f^

S-EO	16	61.35 ± 0.58^h^	12.58 ± 1.11^c^	7.10 ± 3.79^b,c,d^
	32	65.90 ± 0.39^i^	26.25 ± 0.43^a^	10.59 ± 2.79^b,c,d^
	64	73.53 ± 2.08^j^	52.82 ± 0.26^e^	11.18 ± 3.11^d^
	128	94.72 ± 0.84^e^	77.81 ± 0.20^f^	24.93 ± 2.23^a^

Experiments were performed independently at least three times, and the results were expressed as mean ± standard deviation (SD) values. ^a-j^Different letters in the same column indicate a significant difference (*p* < 0.05).

## Data Availability

The data used to support the findings of this study are available from the corresponding author upon request.
